# Using passive Wi-Fi for community crowd sensing during the COVID-19 pandemic

**DOI:** 10.1186/s40537-022-00675-3

**Published:** 2023-01-17

**Authors:** Miguel Ribeiro, Diogo Teixeira, Pedro Barbosa, Nuno Jardim Nunes

**Affiliations:** 1 ITI/LARSyS, Instituto Superior Técnico, Lisbon, Portugal; 2 Mathematics, Instituto Superior Técnico, Lisbon, Portugal; 3 DEI, Instituto Superior Técnico, Lisbon, Portugal

**Keywords:** Passive Wi-Fi, Telecom data, Cellular tower association, Forecasting, COVID-19 mobile application

## Abstract

Sensing passersby and detecting crowded locations is a growing area of research and development in the last decades. The COVID-19 pandemic compelled authorities and public and private institutions to monitor access and occupancy of crowded spaces. This work addresses the detection of crowds in points of interest (POI) by using a territory grid analysis categorizing POIs by the services available in each location and comparing data gathered from a community passive Wi-Fi infrastructure against mobile cellular tower association data from telecom companies. In Madeira islands (Portugal), we used data from the telecom provider NOS for the timespan of 4 months as ground truth and found a strong correlation with sparse passive Wi-Fi. An official regional mobile application shows the occupancy data to end-users based on the territory categorization and the passive Wi-Fi infrastructure in POIs. Occupancy data shows historical hourly trends of each location, and the real-time occupation, helping visitors and locals plan their commutes better to avoid crowded spaces.

## Introduction

During the COVID-19 pandemic, it is essential to know the places and locations where many people are present and where transmission of the disease may occur. Obtaining this information has been the subject of research in various disciplines for several decades. There are different approaches to collecting this data. On the one hand, we have traditional passage sensors, such as pressure mats, optical beams or gates. On the other hand, we have more ubiquitous approaches, using cameras (thermal and facial recognition), wireless signals (Bluetooth or Wi-Fi), or crowdsourced mobile applications. Several mobile applications (e.g. Google Maps) and in-situ devices (e.g. info screens) have integrated information about visitor frequency in POIs, such as supermarkets, shops and restaurants.

In this paper, we explore passive Wi-Fi sensing as an alternative data source with advantages in terms of privacy and data protection by creating snapshots of the territory categorisation that consider the types of services or institutions that exist in each location of locations. Besides that, it shows people’s movements during the 2020 COVID-19 period through their cellular network association records to validate a passive Wi-Fi counter. The cellular data is proprietary, not real-time and only available upon requests. However, with such validation, the real-time passive Wi-Fi detection system can be used as a proxy to infer the locations’ occupation. The combination of real-time passive WiFi data and territory categorisation provides data for a mobile-based recommender system. The infrastructure and the app were used during the COVID-19 pandemic to better inform visitors about which locations and hours are better suited for a visit according to their interests. With this work, we intend to answer the following research questions:

RQ1: Can we visualise the effects of COVID in the mobility data from Wi-Fi and mobile carriers?

RQ2: Can we use passive Wi-Fi data as a proxy for mobility data from mobile carriers?

RQ3: Can we cluster locations and perform forecasts based on the movement activity detected?

## Related work

Previously, relevant research has been carried out in the areas of citizen mobility analysis [1–3]. With the emergence of COVID-19, this area received more attention [4], with several attempts to track individuals on their daily commutes [5] and using contact tracing applications to map encounters between different passerby in public spaces or indoor locations. Motivated by the need to evolve into smart mobility, the internet of things has provided many ubiquitous ways to capture movement data over the last few years. Either by analysing transportation habits [6], smartphone tracking applications [7], call data records (CDR) [1], contact tracing [5] or passive Wi-Fi tracking [8, 9]. Both the areas of passive Wi-Fi [10, 11], as well as cellular mobility [12] have been used to monitor citizens, provide data to decision-makers, or return that data to the communities via interactive interfaces. Smartphones have turned into relevant sensing devices, tagging along on activity data ranging from communications, social networking and contacts, schedules/calendars, entertainment or shopping.

This data gathering makes it possible to practice several spatio-temporal analysis techniques, ranging from crowd controls and scheduling optimizations to detecting mobility patterns and categorizing clusters of behaviours. Besides sensing, these devices can also inform the population or use the collected data to help the citizens in their daily lives by acting as crowdsource mechanisms or user feedback interfaces. We divide this related work into the analysis of cellular network data, followed by an analysis of Wi-Fi monitoring via smartphones, and how collected data has been valuable to inform the citizens about relevant information via mobile applications during the COVID-19 pandemic.

### Cellular network data

When analysing cellular data from mobile carriers, several approaches have been used, the most common being CDR [1, 13], or cell tower movements [1, 14–16]. One of those approaches [17] analysed data from cell tower and WiFi data to apply Hidden Markov Models strategies and try to profile users with anonymised location data to capture the spatio-temporal patterns of each user’s mobility behaviors and use it with statistical models to perform user validations on interactive interfaces. Another study [1] aimed at understanding urban dynamics by using techniques such as Kalman filters to extract mobility patterns and allowing the discovery of mobility hubs, where urban mobility increases or decreases using cellular data. Another approach is to create analytical models [14], where CDR is used to define underlying patterns in the daily life activities of the citizens, detecting through various layers of the model the citizens working hours and classifying people into different classes according to their call habits. Besides the former analysis, from the connections made between people, the platform NextMe [15] tried to create an anonymous location mapping without recurring to the GPS coordinates of the cell towers, simply by making the graphs of the calls and inferring the connections between citizens in space and time by using a novel call pattern recognition. Using a dataset from a real-world user telecom trace resulted in symbolic locations attributed to the users and the analysis of their social relationships in an anonymised space-time system. Cell phone tracking has also been used for monitoring and estimating disease transmission [18, 19]. A particular study [16] has analysed regularly logged cell phone tracking data to map intra-urban transmission of dengue by using irregularly logged cell phone data that miss numerous user movements to perform risk assessments and simulate the uncertainty of the risk. The cellular network has been used to monitor its users, capture epidemiologically-relevant behaviours with mobile phone data, and integrate mobile phone data into decision-making, sending warning and informational messages to citizens of certain regions about risks.

### Cellular geolocation from Google and Apple reports

[20] Following the effort of many technology companies to develop IT solutions to reduce the effects of the COVID-19 pandemic, Google and Apple partnered into the most widely implemented [20] exposure notification protocol. In a first phase, both companies would release APIs that would allow contact tracing apps from public health authorities to work across Android and iOS devices [21]. In a second phase, google and Apple built the bluetooth-based contact tracing directly on their underlying operating systems with the user’s consent of usage [22].

Immediately, a platform was created (currently disabled) to provide reports about the mobility trends of driving and walking, to analyse the mobility activity within each region. Several articles have used this information to analyse the mobility trends and explore the effects of COVID-19 in the population.

The authors in [23] have used the data to identify, quantify and classify different degrees of social distancing, while observing a strong decrease in the infection rate occurring two to five weeks after the onset of mobility reduction.

In Japan, [24] this study has analyzed the behavior changes of four areas based on Google and Apple mobility data reports, showing that the pre-pandemic patterns have been changed significantly during the COVID-19 waves, and also that these changes have differences depending on the urban structure and climate factors. Another study in Japan [25] also looked into this data to evaluate a virus reproduction rate *R(t)*, demonstrating that Apple mobility data is useful for short-term prediction of *R(t)*.

This study [26] has a different approach, in that it combines data not only from Google and Apple, but also from disease statistics from the European Centre for Disease Prevention and Control. It took place in 26 countries during 5 weeks and they integrated the data into a machine learning Gradient Boosted trees approach to explain that the variation in mobility patterns can be explained in 47% by the variation in the disease transmission rates, thus confirming the effectiveness of social distancing interventions in slowing the spread of COVID-19.

### Wi-Fi tracking

Several challenges are faced depending on the type of location, how constrained or how intrusive the system is, and how costly it is to install. Several attempts have looked at Wi-Fi as a means to infer crowd densities. Due to its non-intrusive nature, passive Wi-Fi tracking has been studied and applied in several situations in the past, ranging from university campuses [27, 28], and tracking spectators in football stadiums [29], to small cities [11] and public transportation [30, 31]. The motivations of these studies are diverse, some look at energy waste on scanning methods [27, 32], others focus on realistic facility management and planning [33], and even analysing crowding factors and flock detection [34], waiting times in public transport [35], frequent paths [36], while also inferring social information, like the popularity of events [29].

It has been previously applied ubiquitously in urban locations and points of interest (POI), using methods for crowd detection [34, 37, 38]. In the last year, with the expansion of COVID-19 and the concerns for public locations crowd monitoring and control, this technology emerged evermore with many practical applications. Similarly to contact tracing COVID-19 tracer [39] attempted in a university to detect proximity calculation via RSSI (Received Signal Strength Indicator) and a location similarity coefficient to infer close contacts to other people that may be tested positive afterwards. Wi-Sneeze [40] is another system that uses Wi-Fi signals to analyse the presence of sneeze particles in the environment analysing the Doppler frequency of the signals for changes provoked by sneezed particles. A similar approach was done by trying to detect periodic human movements used for coughing or breathing speed in controlled environments [41]. On the field, occupation estimation in public spaces has also been done via a passive Wi-Fi infrastructure, while having surveys to inform and collect passerby feedback of the information presented [8]. All these emerging solutions exploit the ubiquitous nature of passive Wi-Fi, the lack of user intervention and the need to monitor occupation or behaviors on commonly used spaces.

### COVID-19 related mobile apps

Mobile applications have been used as effective solutions to health monitoring and diagnosis [42–44]. With the COVID-19 emergency and the need to track close contacts with individuals that tested positive, there was an opportunity to deploy digital contact tracing using Bluetooth and other techniques with attention to efficacy, privacy and security. Solutions rapidly emerged from Apple (iOS),^1^ Google (Android)^2^ and others [45, 46] as background services to perform that tracking and making APIs available to developers to implement their own usage of that anonymized data. Many countries and regions adopted these solutions, implementing their applications for digital contact tracing and informational purposes, including the Australian CovidSafe [47], Covid Watch by MIT Safe Paths, SwissCovid [48], Austrian Stopp Corona App, Portugal’s StayAway COVID [49], with many other countries and regions that followed with their applications [5]. Privacy concerns quickly became aware of the users, and the population [50, 51], concerning data protection, sharing personal information with others and having that information on many privately owned databases, which also led to some unwillingness to use those applications [52].

## Location and datasets

To develop this research, we resorted to three data sources ranging from 2019 to 2020: two related to people’s mobility across different locations (telecom data and passive Wi-Fi) and a third one that categorizes the services available in those locations (OpenStreetMap).

### Location

**Fig. 1:**
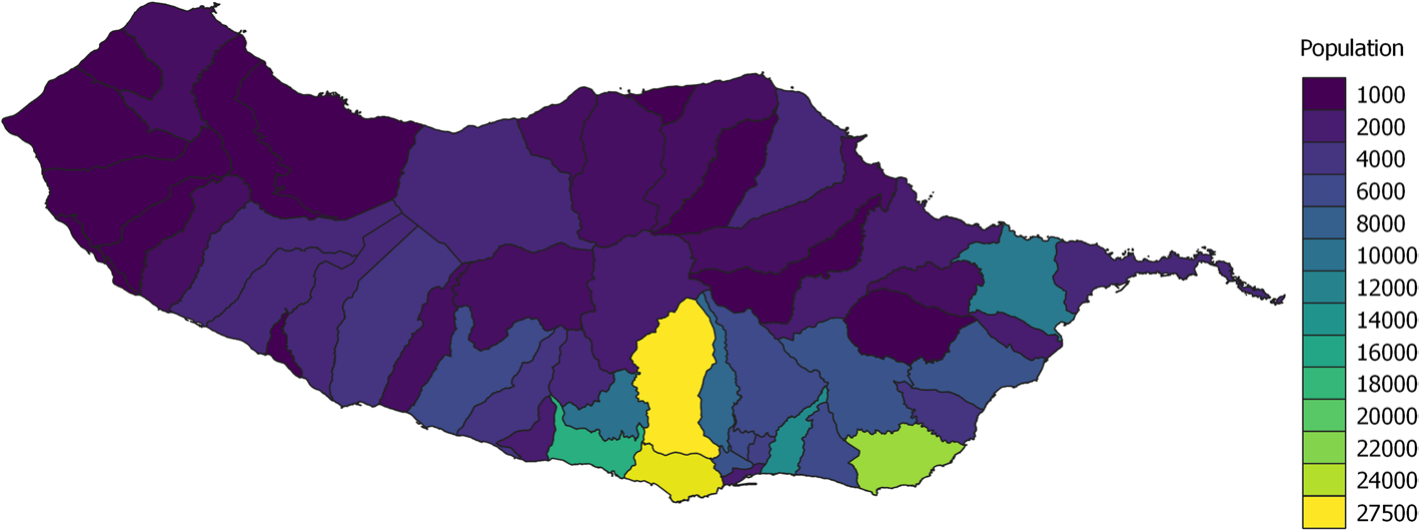
Population per parish. Population per parish in the island showing the parishes with more population density

This study was conducted in Madeira Islands, a medium-sized Portuguese archipelago situated in the north Atlantic, southwest of Portugal’s mainland (1 h30 min flight from Lisbon). The southerly marine position of Madeira Islands renders a warm year-round subtropical climate which, together with striking landscapes and beaches, makes these Islands a popular tourist destination. Tourism is an important sector in the region’s economy, contributing to more than 25% of the region’s GDP and providing support throughout the year for commercial, transport and other activities and constituting a significant market for local products. The two populated islands are Madeira and Porto Santo, comprising a total of 270.000 inhabitants, the clear majority (98%) in Madeira Island. Madeira is just over $700\,\hbox {km}^2$ but holds the third-largest metropolitan area in Portugal around its capital, Funchal, with over 110.000 inhabitants, with a population density of $1\,469.36\,\hbox {hab./km}^2$. Figure 1 shows the population density per parish in the island.

### Dataset—Telecom data

The telecom data contains anonymized counts of cell phone registrations into cell towers. These counts were exceptionally provided for anonymized analytics purposes by a national telecommunications company in the context COVID-19 pandemic, consisting of an origin/destination matrix of all parishes and the counts of cell tower transitions by the same cell phone ID time spans of 2 h. This dataset ranged from February 2020 until May 2020, adding up to 4 months of data. In addition to that, it also differentiates between the cell phone counts with national affiliation vs foreign (roaming). The market shares already extrapolate all the counts provided to represent the movements of all telecom companies operating in the region, thus representing the whole population.

### Dataset—Passive Wi-Fi/Beanstalk

The visualizations depict the data collected from a passive Wi-Fi platform [53, 54]. The research team designed this infrastructure originally to support a community-based Wi-Fi tracking system to understand mobility at scale. The original system provided several interactive dashboards [36, 55, 56] to help communities quickly run a systematic analysis of tourists’ mobility patterns in the destinations, contributing to new ways of visualizing spatio-temporal mobility data.

**Fig. 2:**
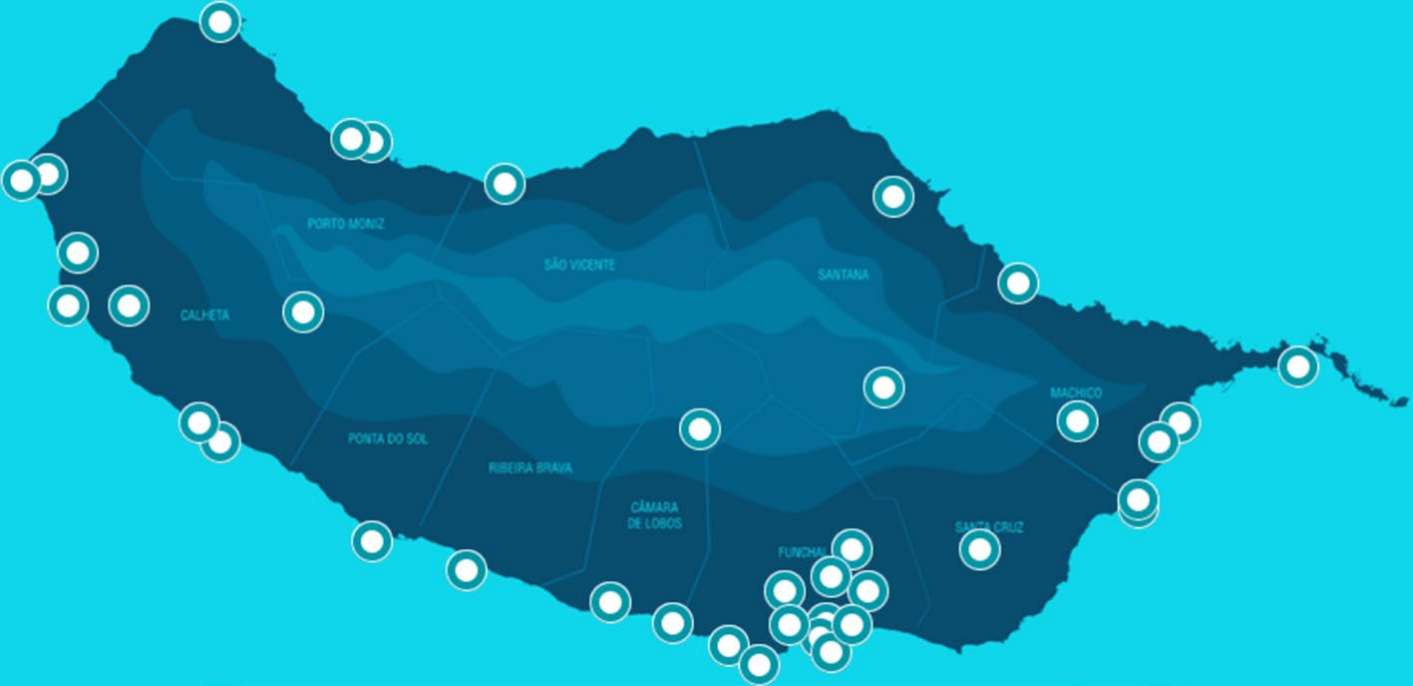
Passive Wi-Fi locations around the island. Passive Wi-Fi locations around the island

This system is composed of 82 Wi-Fi routers spread across all municipalities of the region, as shown in Fig. 2 deployed in the wild, in unsupervised public locations. The data is collected in real-time by Wi-Fi routers spread across multiple sites and then sent to a central server. The information includes a POI identifier, a timestamp and an anonymous identifier (based on the MAC address) of each Wi-Fi enabled device present in a radius of approximately 80 m from the router (depending on the location and obstacles). The processed results are made available via an API that allows the development of standalone implementations, thus providing real-time information about the number of devices detected in the last hours, location meta-data, and historical hourly data that compares to the current counts.

### Dataset—OpenStreetMap services/infrastructures geodata

To categorize an area, we need to understand the type of establishments available nearby. This data source aims to categorize each island location by pulling meta-data from OpenStreetMaps about the services available in the region. We use OpenStreetMap to get that data by downloading all the POIs for the island. The data was collected in April 2020, composed of 730 089 records, where each record had a longitude, latitude and type of building. We grouped the many types and subtype locations provided by OpenStreetMaps into the categories described in Table 1.

**Table 1:** Location Categories grouped from OpenStreetMaps

POI Group	Count	Description	Examples
Commercial	887	Commercial/community establishments	Shop, Supermarket
Community	528	Places that provide services to the community	Sport Centre, Picnic site
Educational	217	Establishments regarding education	Kindergarten, School, University
Entertainment	238	Places related to attractions	Aquarium, Museum, Theatre
Financial	167	Establishments related to money	ATM, Bank
Government	201	Establishments owned by the Government	Embassy, Courthouse, Police
Healthcare	213	Places that provide medical services	Dentist, Pharmacy, Hospital
Living	8425	Places with houses and apartments	Apartments, Residential, Dormitory
Sustenance	1550	Establishments with food and drinks	Bakery, Bar, Cafe, Pub, Restaurant
Tourism	1103	Places with tourism interest	Hotel, Information, Viewpoint
Transportation	1242	Any kind of transportation services	Bus Station, Taxi, Car rental

## Data processing and results

A stepwise process was conducted to analyse the data. In the first analysis, we grouped and mapped the territory categorisation data to understand better the parishes and locations under study. Then we detected that the Wi-Fi data contained several outliers that needed to be cleaned up before proceeding to the remaining analysis. After that, we compared the Wi-Fi data against the telecom cell movement records, the infrastructure geodata, and the telecom data from the Wi-Fi counts. Then, we analysed the Wi-Fi data in terms of clustering and forecasting the people’s presence from the Wi-Fi counts. Finally, we provide a use case of how this information is already being used in a mobile application to provide the citizens with these processed results to help them make informed choices of the times of day and locations to visit in a COVID-19 environment.

### Territory categorization

**Fig. 3:**
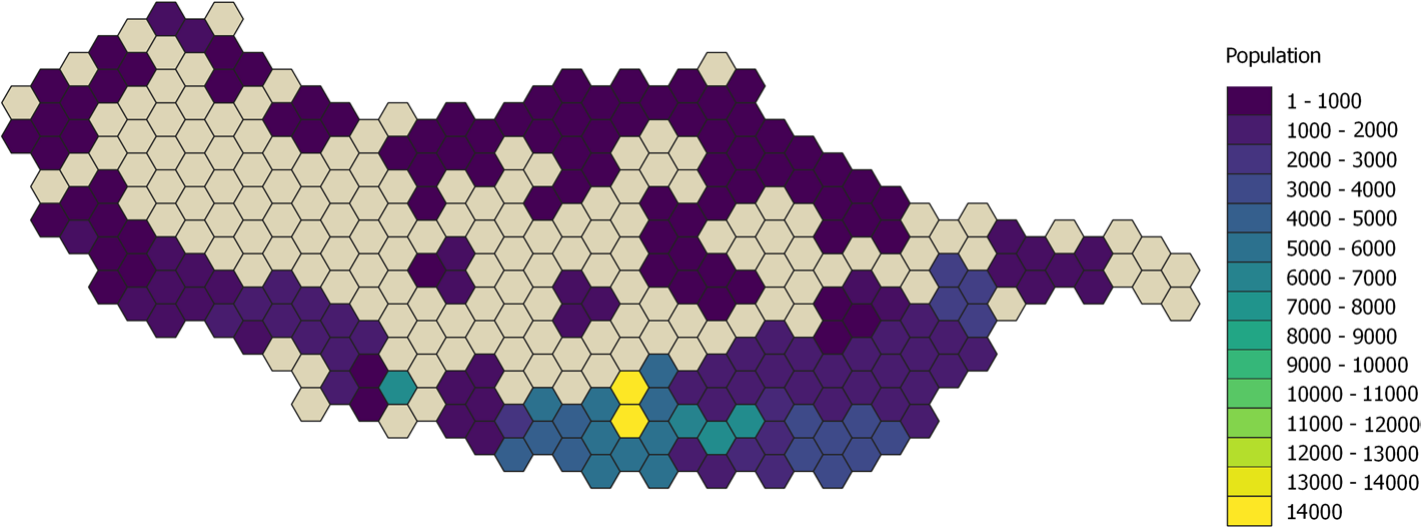
Calculated population per hexagon. Colorized map showing the calculated population per hexagon of the territory grid

**Fig. 4:**
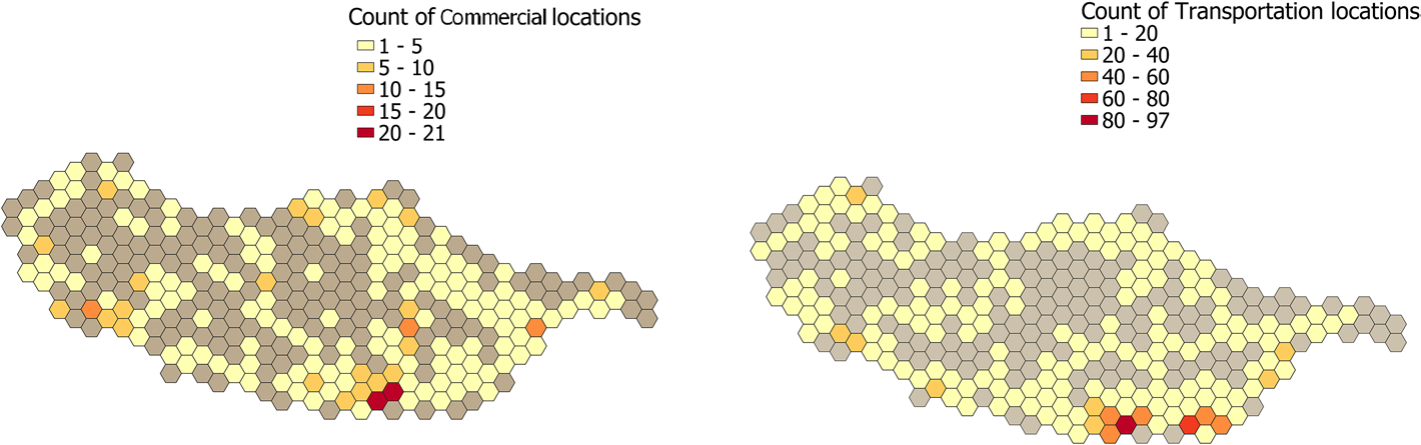
Hex-Grid with commercial and transportation distribution. Hex-Grid with commercial (left) and transportation distribution (right) maps of the region

Using the data collected from OpenStreetMaps, we divided the island’s territory into hexagons that coincided with the areas covered by the passive Wi-Fi data. Each hexagon has an area of 2.7 km$^2$, and in Fig. 3 we first represent the population distribution on the island to get a sense of the areas envisioned to have more services available to the population. The population per hexagon was calculated based on the population per parish (Fig. 1) then distributed according to the areas of the map that had more services/infrastructures of the type “living”. Then in Fig. 4 we present an example of two categories of table 1. From these two figures, we can observe that the maps from the population and the services partially overlap as expected. The maps in Fig. 4 differ due to their inherent characteristics of not all locations being covered by public transport.

**Fig. 5:**
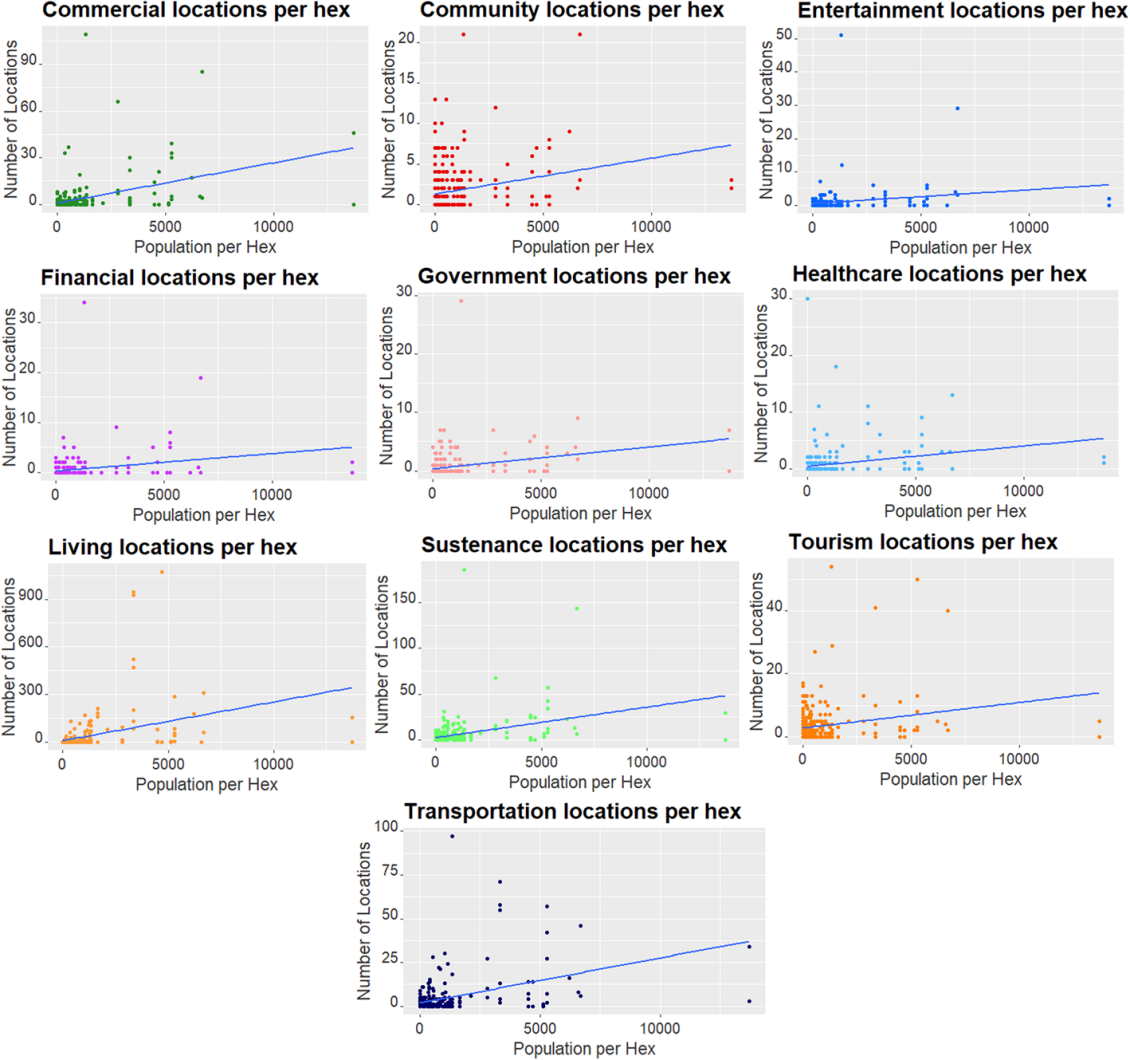
Services listed per hexagon vs population. Number of services listed per hexagon against the population, for each category

We then represented each category by the number of services listed per hexagon against the population that lives there. Figure 5 shows the scatter plots where we can observe higher correlations between the population and the services in the categories such as entertainment, commercial, financial, and sustenance. In comparison, we can find more dispersed data in healthcare, tourism and transportation and government services. This dispersion shows that areas with a higher concentration of tourism are not those where the general population lives. Or that the public transport passes through many locations with a sparse population, connecting more distant cities.

### Outlier detection

With the passive Wi-Fi data, due to the nature of the deployment on 3rd party infrastructure. Some Wi-Fi routers fail or get disconnected, and that creates missing values or abnormalities in the timeline, which can hinder some analyses which require uninterrupted datasets, so we performed the following steps to pre-process the data and remove outliers:

a) Based on the number of missing values, we searched for a period of at least 1 year (before 2020) 25 routers were selected, from May 2018 to December 2019, which have under 20% of missing data. We ensure good data is used for the data analysis, preventing holes in the timeline with this criteria.

b) Outliers Detection: To detect the outliers in each router data, we used a rolling Z-score with a 30-days window to classify the observation in data as normal values or outliers. This method was based on the z-score value of an observation. If that value is superior to three, that means that the observation is three standard deviations away from the average and can be considered an outlier (since 99.7% of normal data is within three standard deviations from the mean).

c) Outliers Correction: Outliers that with a large amount of missing data were replaced by the median (since the median is robust to outliers, unlike the average) of data grouped by router, time of day and weekend flag. The remaining outliers are replaced by a rolling z-score of 30 days: $x_{t} = {{\overline{x}}}_{[t-15, t+15]} + 2 \sigma _{[t-15, t+15]}$, where ${{\overline{x}}}_{[t-15, t+15]}$ and $\sigma _{[t-15, t+15]}$ is the rolling average and standard deviation from the 30-days window centered around $x_{t}$. We choose to sum 2 standard deviations to the mean to guarantee that will become acceptable values in the data.

d) Missing Values: In this case, we grouped the data by a router, time of day and weekend flag and then computed the mean using a 90-days centred rolling window. Because the data still has missing values, they can cause some of the averages of these groups to be invalid values. To prevent computing with invalid values, we use the 30-days rolling average of the entire data by the router when this happens.

**Fig. 6:**
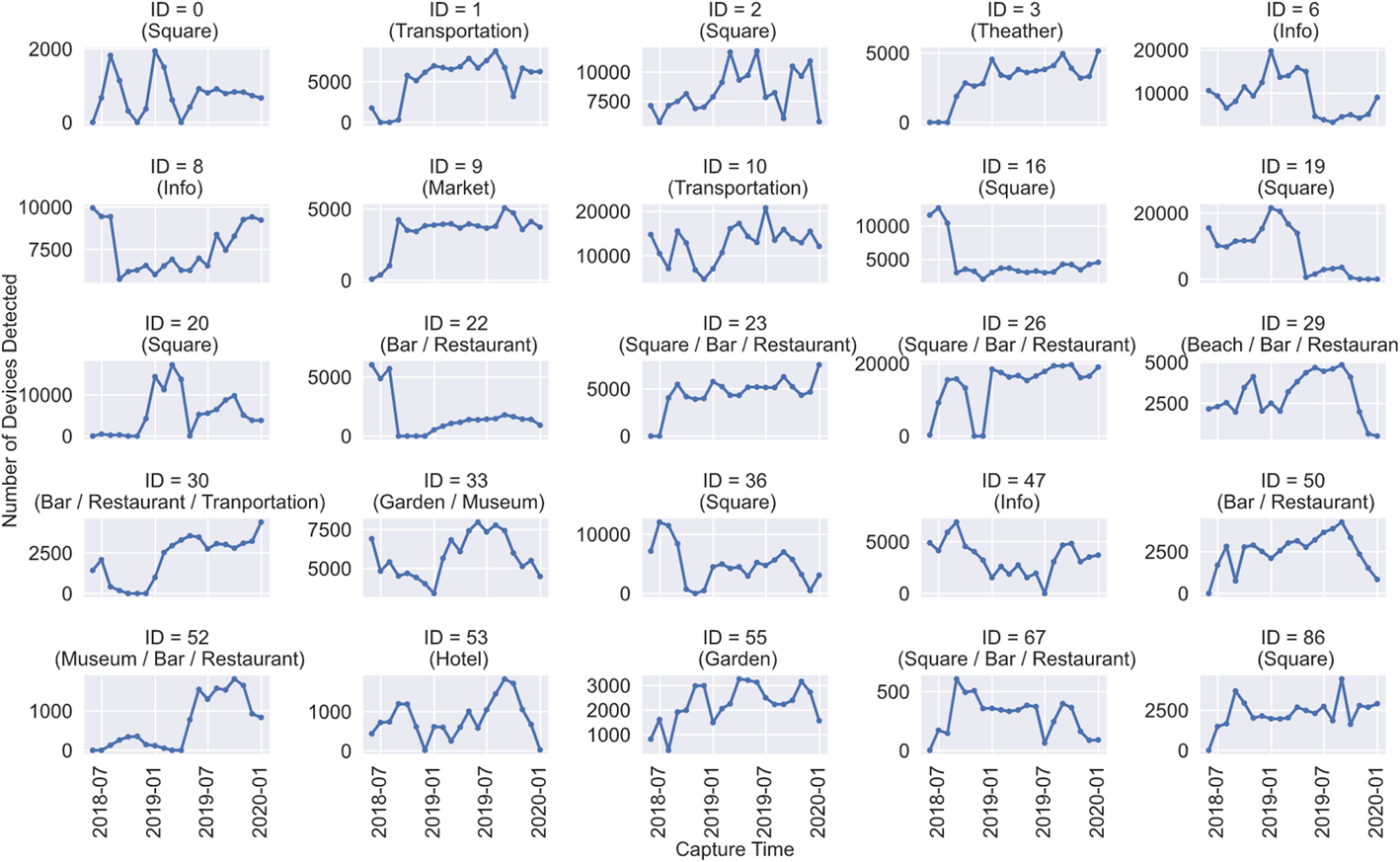
Monthly average of detected devices. Monthly average of daily number of devices detected by each passive Wi-Fi router

After pre-processing the raw data, such as attenuating the outliers and inputting the missing values, Fig. 6 represents the dataset of the selected 25 routers between May 2018 to December 2019, with a monthly average of the daily counts. We can see that the time series has very distinct shapes, such as peaks and falls at different months. However, there seems to be a consistent increase in the number of devices detected in the summer months, showing a general trend of summer activity towards most of the time series, as expected.

### Wi-Fi data against territory categories

**Fig. 7:**
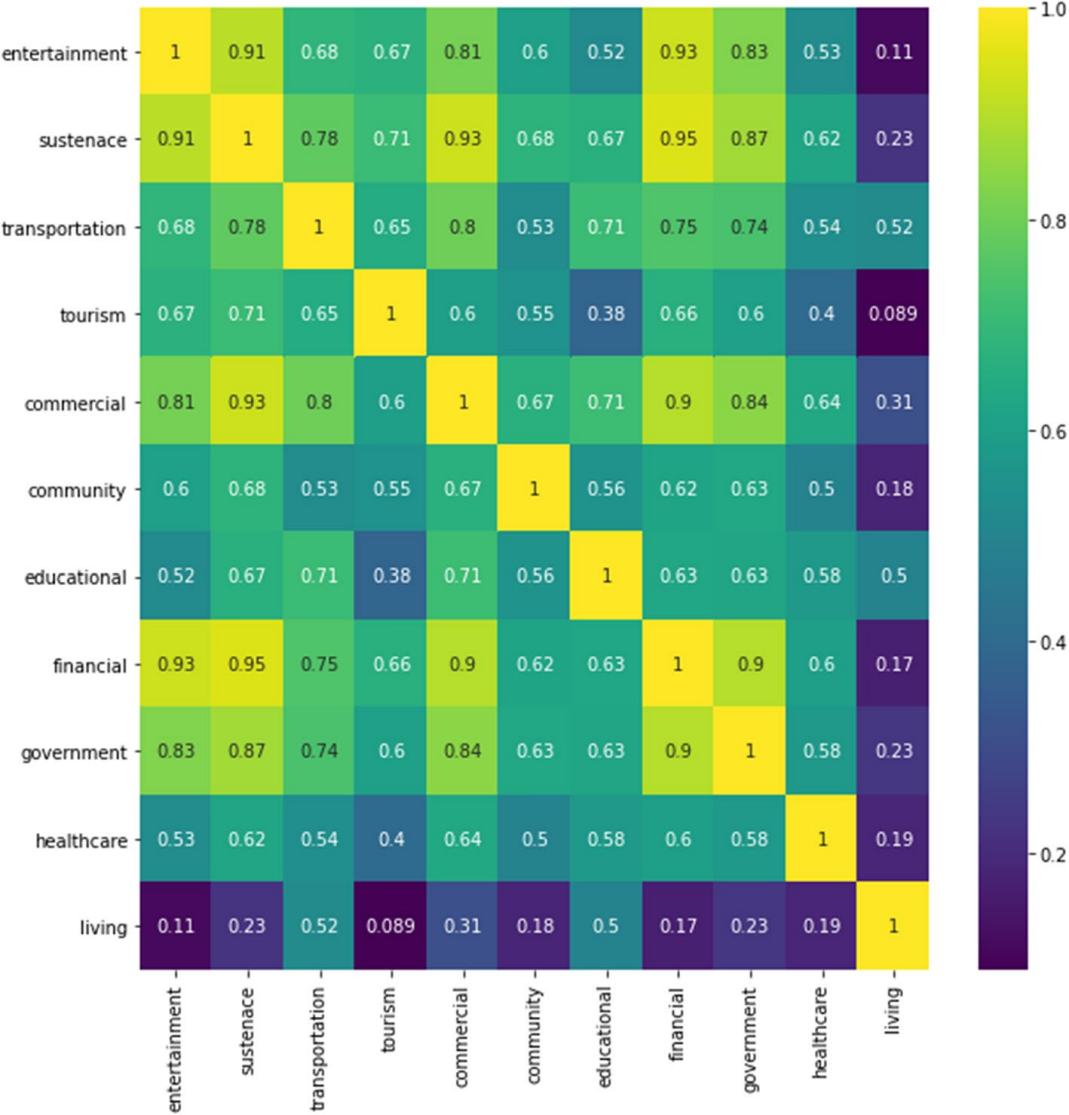
Self-correlation of services around the island per hexagon. Self-correlation of services around the island per hexagon

Another interesting analysis is to correlate the territory categories among datasets. In this analysis, we first tested the self-correlation of the number of services available around the island, depicted in Fig. 7. It shows high correlations between some distinct groups of categories, where we can highlight the dependencies between sustenance, entertainment, tourism and finance (possibly due to ATMs). Then between sustenance and commercial and financial and government. Among these groups, there is a strong connection between government and finance. On the other hand, we can once again see the effect of very little correlation between tourism and living, for instance, where the higher connections of living are with education and transportation. As expected, another shortage of services present in the same areas is tourism and education.

**Fig. 8:**
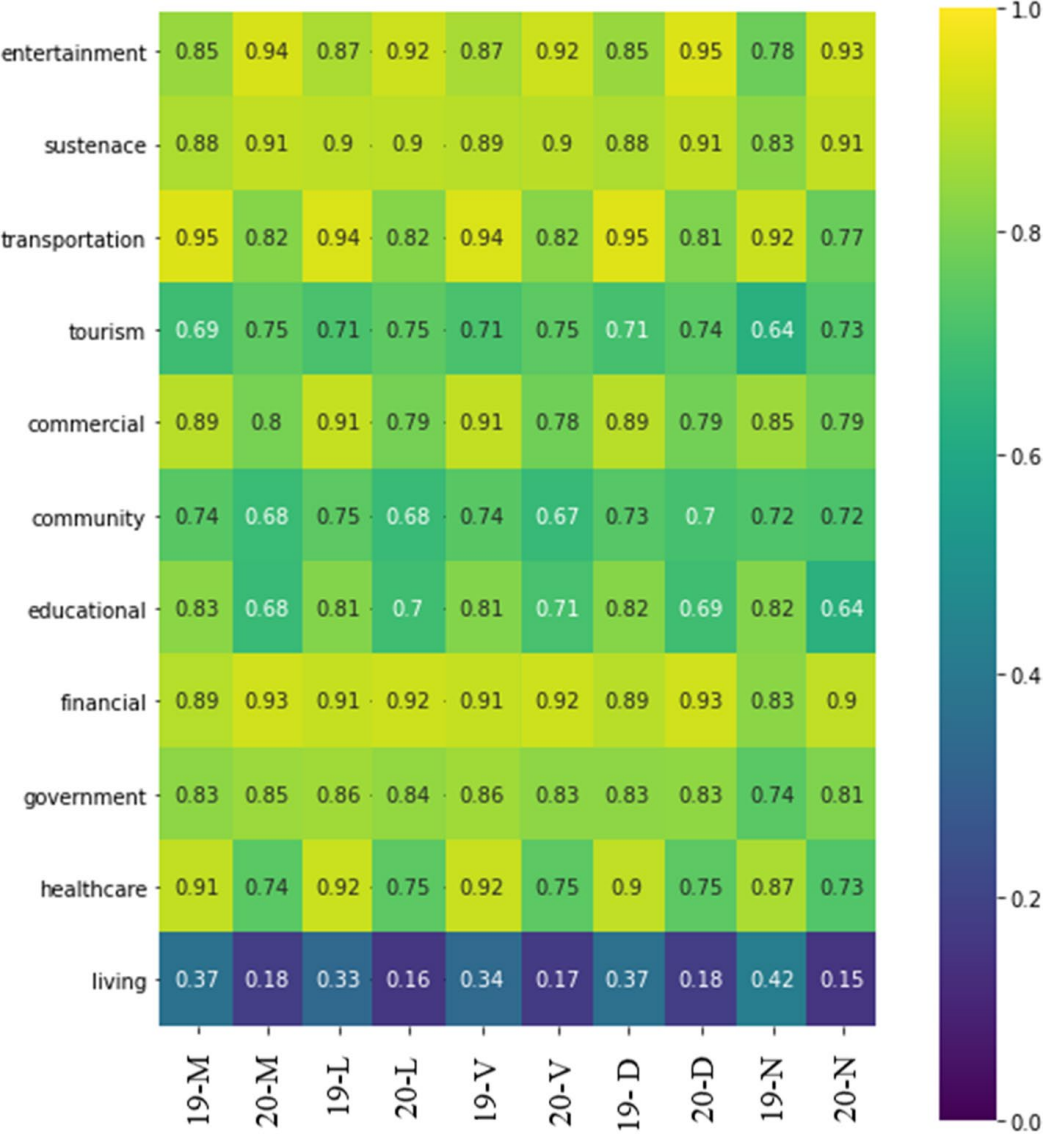
Wi-Fi counts vs establishment counts. Wi-Fi counts vs establishment counts by category in each hexagon (M: Morning; Lunch-Living; Evening-Ver; D: Dinner; N: Nightlife)

The next step was to see some correlation between the numbers of devices detected by the passive Wi-Fi infrastructure from 2019 and 2020, grouped by hexagon, against the number of services available in each hexagon. Figure 8 depicts the results for different times of the day of Wi-Fi counts. We can observe very high correlations between these two variables. The horizontal axis shows the contrast between 2019 and 2020, with slight differences in transportation, education and commercial, which decreased due to the effects of the COVID-19 confinement periods. The values of the category living seem to be very low. One explanation is that passive Wi-Fi routers were installed in locations deemed as points of interest, thus not representing well the areas of residences, apartments or dormitories.

### Telecom mobility data

**Fig. 9:**
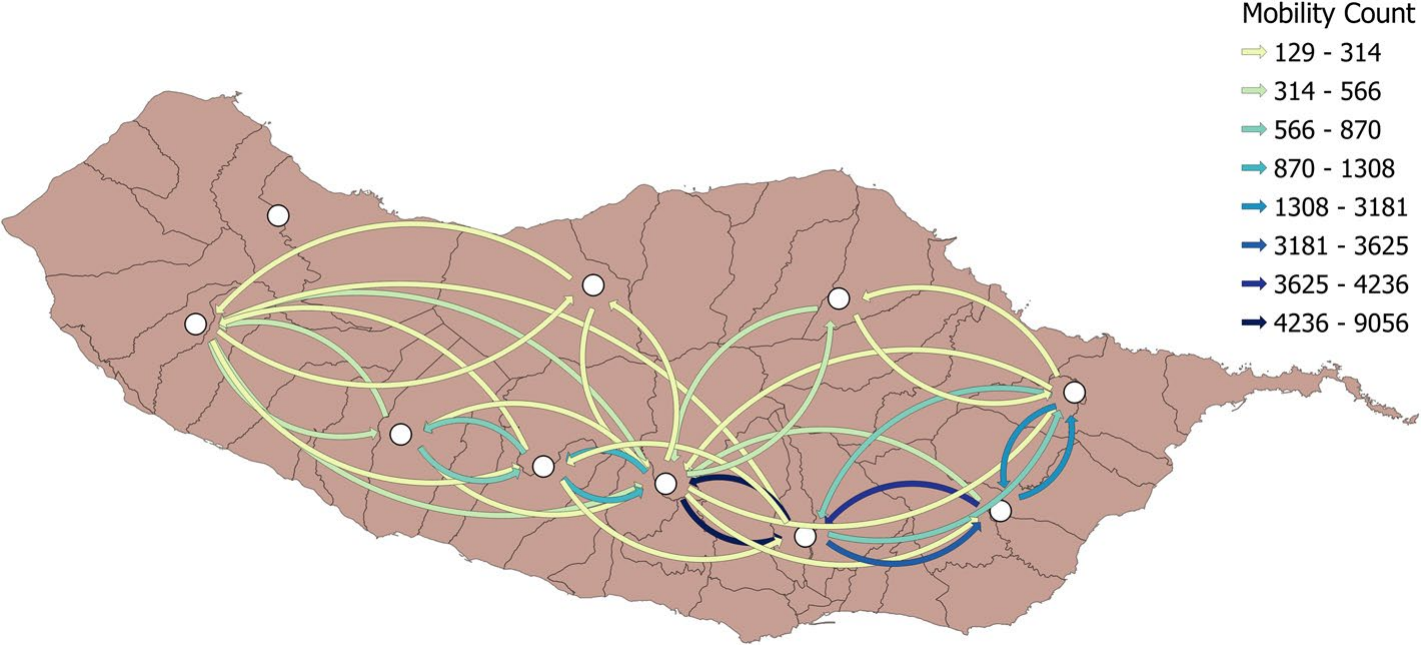
Cellular associations by municipality. Cellular tower associations changes/movements by municipality from the telecom dataset

The telecom data provided mobility counts for cellular tower associations to the municipality granularity, thus not directly comparable to the Wi-Fi data or population associated with the previously presented hexagons. It allowed, however, to compare its connections between municipalities of the island, checking the mobility and the most prevalent links that existed. We represent those movements in Fig. 9, and we can see the mobility counts focus more on three main municipalities on the lower right of the map that corresponds to the location of the airport and the two most populated cities of the island. Although some movements occur between municipalities that are not adjacent to each other, these can be possible due to the location of the cellular towers, which do not necessarily follow the territorial divisions of the municipalities.

### Comparing telecom data with WiFi

**Fig. 10:**
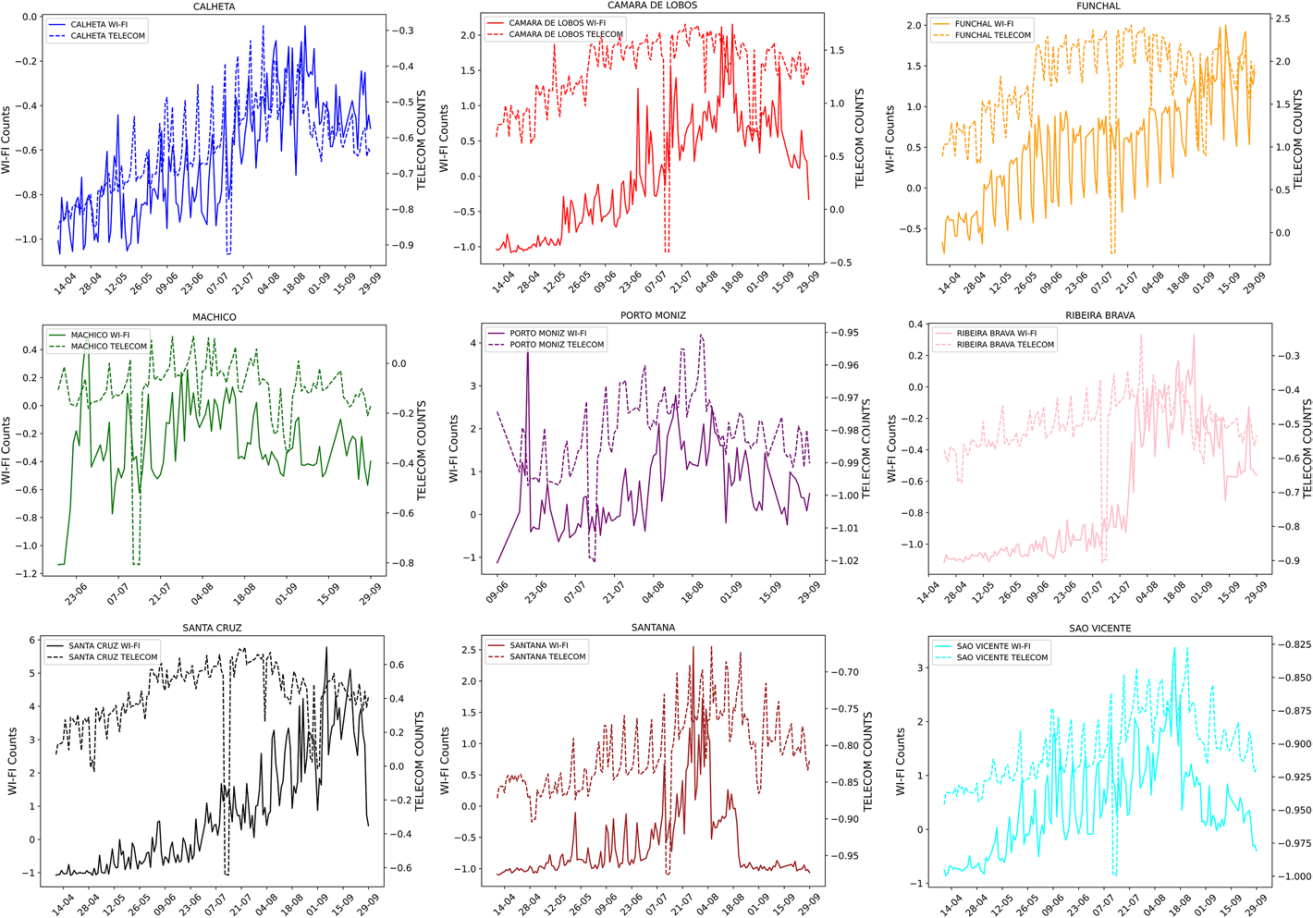
Wi-Fi vs telecom by parish. Weekly comparison between Wi-Fi data and telecom data by parish

**Fig. 11:**
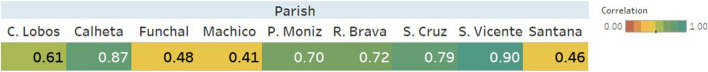
Wi-Fi data vs telecom correlation. Weekly correlation between Wi-Fi data and telecom data by parish

With the deployment of the passive Wi-Fi infrastructure in the wild and limited possibilities of obtaining systematic ground truth for each POI, we gathered the municipality’s Wi-Fi data and the more reliable telecom data. Our goal was to use that as a proxy to infer the reliability of the passive Wi-Fi infrastructure. The telecom data included various features related to the tower association movements, and thus to perform this comparison, we applied the PCA (Principal Component Analysis) to both the datasets of the hourly device counts of the Wi-Fi dataset and the telecom dataset. We depict the results in Fig. 10, where we can observe the two series plotted in time for each municipality. While the data for some municipalities do not follow each other, the majority follow the same trends with activity peak increases and decreases. We also show those correlations in Fig. 11, where even the lower scoring municipalities have values very close to 0.5, and the highest reaching 0.9. Upon locating these municipalities on the map and seeing the location of the passive Wi-Fi routers, we can conclude that in the municipalities with a lower population (more concentrated) the Wi-Fi device detection is a good indicator of the activity of people in the area. In contrast, in municipalities with a more dispersed population or fewer Wi-Fi routers, the Wi-Fi counts do not represent the totality of the people activity.

### Clustering and forecasting

**Fig. 12:**
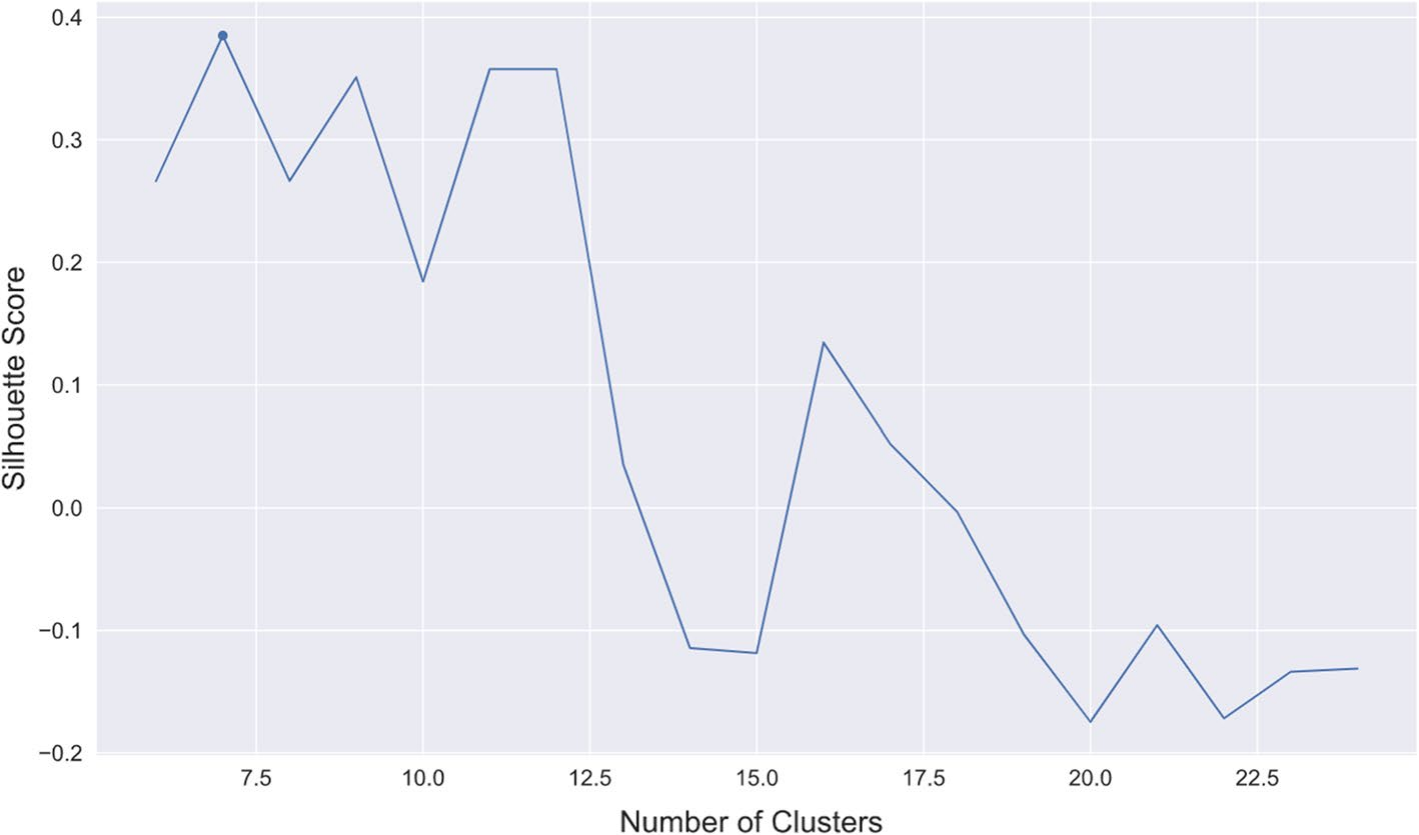
Silhouette score. Silhouette score used to determine the number of clusters

This analysis tried to find some clusters of locations with similar activity based on the Wi-FI device counts. To reduce the number of series to forecast, we clustered the routers and then used a top-down approach to hierarchical forecasting. First, we tried some time series Similarity Measures, such as DTW (Dynamic Time Warping), PCA and Mahalanobis distance-based measures, since this would be appropriate for clustering time series. This proved ineffective in clustering the routers since, despite the high silhouette score obtained, most routers were all in the same cluster. As a solution, we tried to create several features from the data that could be used to perform a more classical approach to clustering. The selected features that performed the best in terms of silhouette score (Fig. 12), were the means of each router when grouped by weekday and time of day. We applied PCA and used the top three principal components. Then we used approaches like hierarchical and spectral clustering and k-means, selecting seven clusters based on silhouette scores (score of 0:4) and spectral clustering. Then the routers in each cluster were summed by day, obtaining a data set of seven time series with daily frequency.

**Fig. 13:**
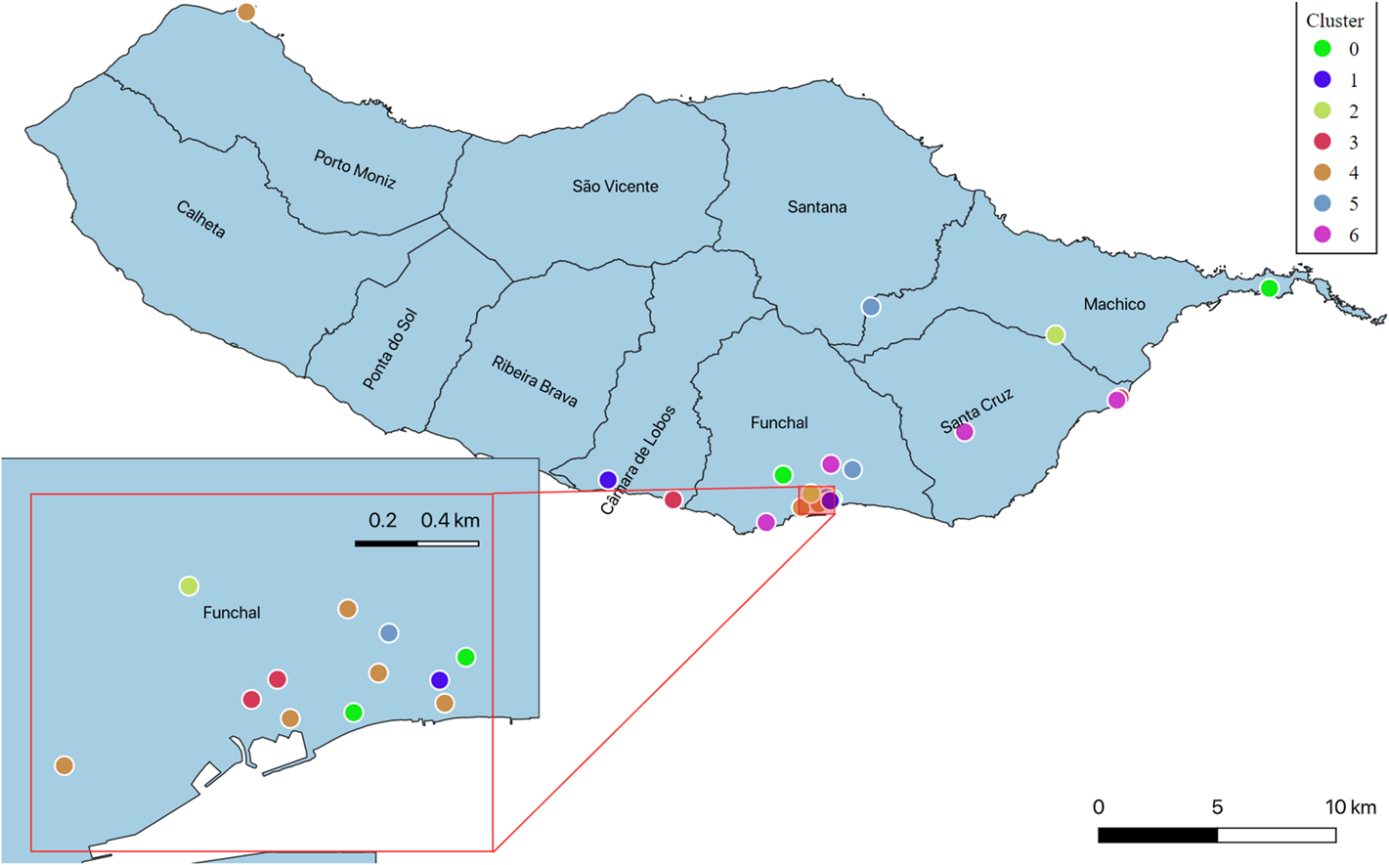
Clustered Wi-Fi map. Wi-Fi router map grouped by cluster

In Fig. 13, each router is aggregated by cluster (see legend) and represented on a map of Madeira Island to be able to visualize how these clusters relate in space.

Using the clusters from the previous section, we applied several methods to try and forecast data taking into account the different clusters: Vector AutoRegression (VAR), Vector Moving Average (VMA), Vector AutoRegression Moving Average with exogenous variables (VARMAX). However, to forecast using VARX, VMA, and VARMAX models, the time series needs to be stationary and not co-integrated. This means that it shouldn’t have a significant trend or seasonality component. To identify that, we used an autocorrelation plot or statistical test such as:

- KPSS test—tests if the univariate time series is trend stationary;
- ADF—test for the presence of unit root in the time series, i.e., test if the univariate time series is non-stationary;
- Johansen Cointegration test—test if a multivariate time series is cointegrated.

**Fig. 14:**
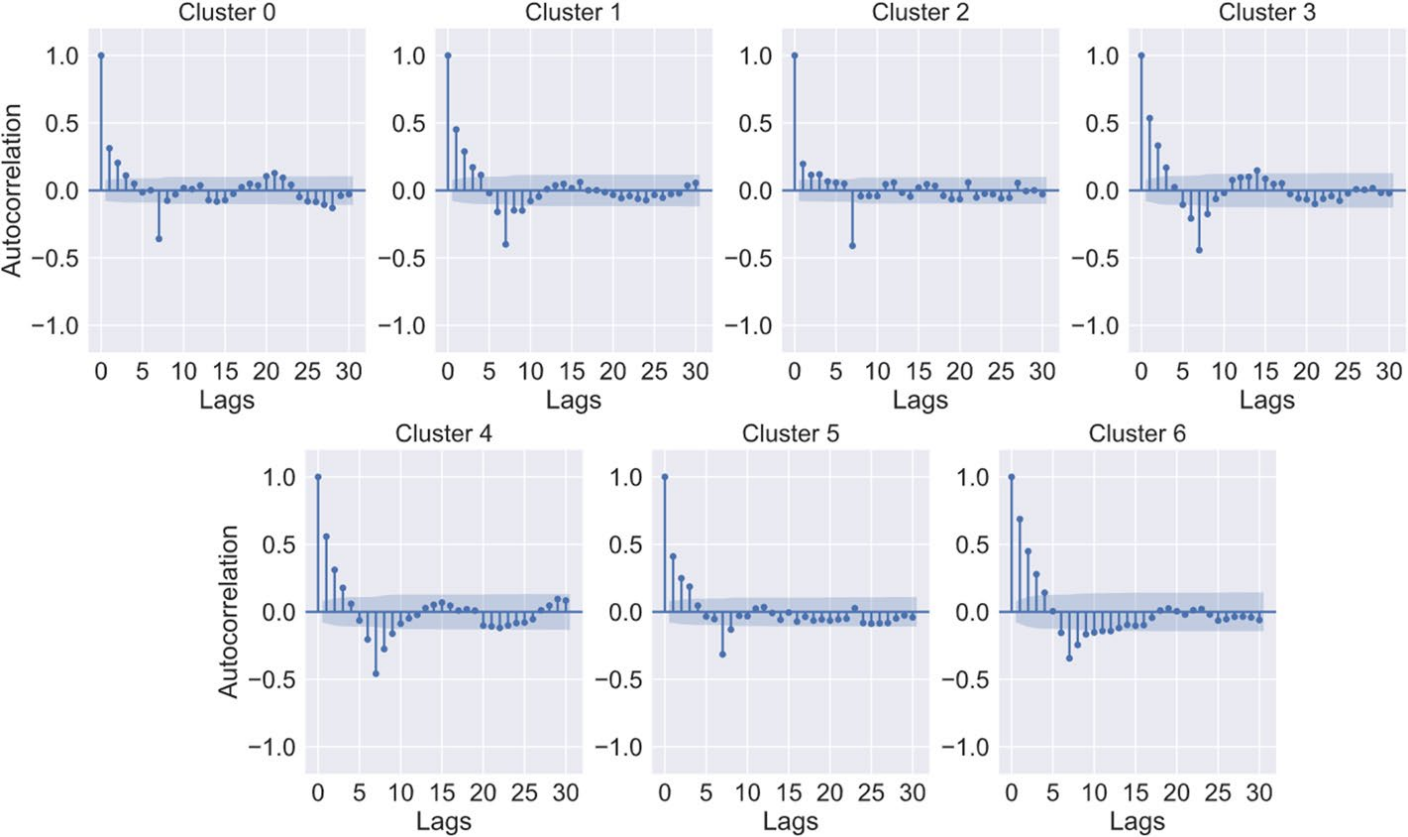
Multivariate time series autocorrelation. Autocorrelation function of multivariate time series, after applying a logarithmic transformation

In Fig. 14, we show the autocorrelation function of multivariate time series, after applying a logarithmic transformation to the data and differencing to remove seasonality, is represented here until lag 30, this is, the correlation between observations *y*(*t*) and *y*(*i*) where $i=0,1,\ldots ,30$. It’s possible to see that the ACF falls within the confidence interval at most lags, meaning that it is not significant. There is still some seasonality at lag seven, but it’s very attenuated.

The autocorrelation plots show a seasonality indication of seven days and a trend in this time series. Both KPSS and ADF tests also show significant existence of non-stationarity in most of the time series. So then, the co-integration test needed to be performed, and it showed that there was co-integration between the time series. So the most appropriate model to use would be VECM (Vector Error Correction Model). But some studies show that even co-integrated time series models like VAR, VMA and VARMAX, may perform better in forecasting. So we will test and evaluate all these models. For VAR, VMA and VARMAX models this was the methodology followed is summarized on Table 2.

**Table 2:** Steps taken to model and predict data using VARMAX and VECM methods

VAR, VMA and VARMAX	VECM
1) Apply logarithmic transformation to the data, to guarantee that all forecasts are positive;	
2) Apply differencing of 7 to remove seasonality $z_{t} = y_{t} - y_{t} - 7$;	2) Train VAR model to choose lag order with AIC;
3) Apply differencing of 1 to remove trend (if needed) $z_{t} = yt - y_{t} - 1$;	3) Select cointegration rank, using the Johansen Cointegration test;
4) Train model (60% of data) and choose lag order based on AIC	4) Train VECM model with the selected lag order and cointegration rank using train set (60% of data);
5) Test best model (lowest AIC) using the test set (40% of data);	5) Test model using the test set (40% of data);
6) Test the best model (lowest AIC) using the test set (20% of the data)	

### Forecasting results

a) With VARX, we obtained AIC of $-$24.56 for lag order of 8 and with the exogenous features: holiday flag (if it’s a holiday in Portugal or not and month 10 (regarding if it’s October or not), selected by feature selection with AIC. When evaluating on the test set, we obtained 17.70% of symmetric mean absolute percentage error on average, considering all clusters. b) With VARMA, we obtained AIC of $-$2110.97 for lag order of 8 (AR components) and 1 (MA components). We got 17.40% of symmetric mean absolute percentage error on average when evaluating the test set, considering all clusters. The residuals of the VARMA and VARX models did not respect the whiteness assumption, invalidating one of the assumptions about the error term of these models, which may be due to the existence of co-integration in the multivariate time series. But as some authors recall, when the final objective is forecasting, this may not affect the point forecasts. c) For VECM, we obtained a lag order of 8 to the VAR component and then estimated a co-integration rank of 1. Unexpectedly, the VECM forecast gave the worst predictions since, when evaluating the test set, we obtained 23.81% of symmetric mean absolute percentage error on average, considering all clusters.

**Fig. 15:**
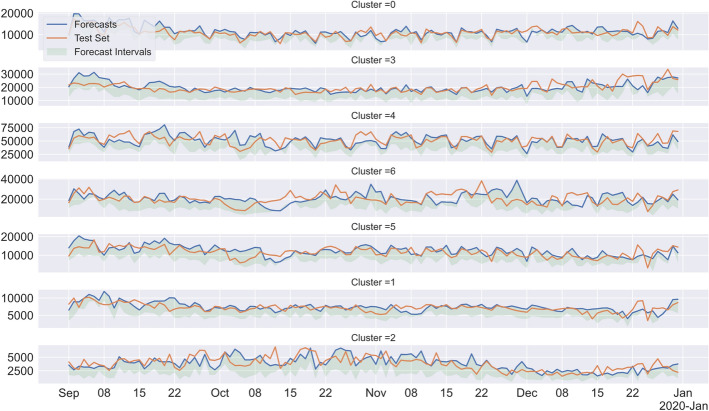
VARX Forecasting. Forecasting data using VARX for all clusters

In Fig. 15 we can see the forecasts and test set, along with the confidence intervals for the forecast. For ease of visualisation and comparison, when forecasting with the VARX model, using a lag order of 8 (selected with AIC), meaning that each observation is predicted based on the previous 8 observations, and also using the exogenous features of holiday flag—if it’s an holiday in Portugal or not—and also a binary variable indicating the month of August. We obtained 17.79% of symmetric mean absolute percentage error on average when evaluating the test set, considering all clusters.

## Mobile application

**Fig. 16:**
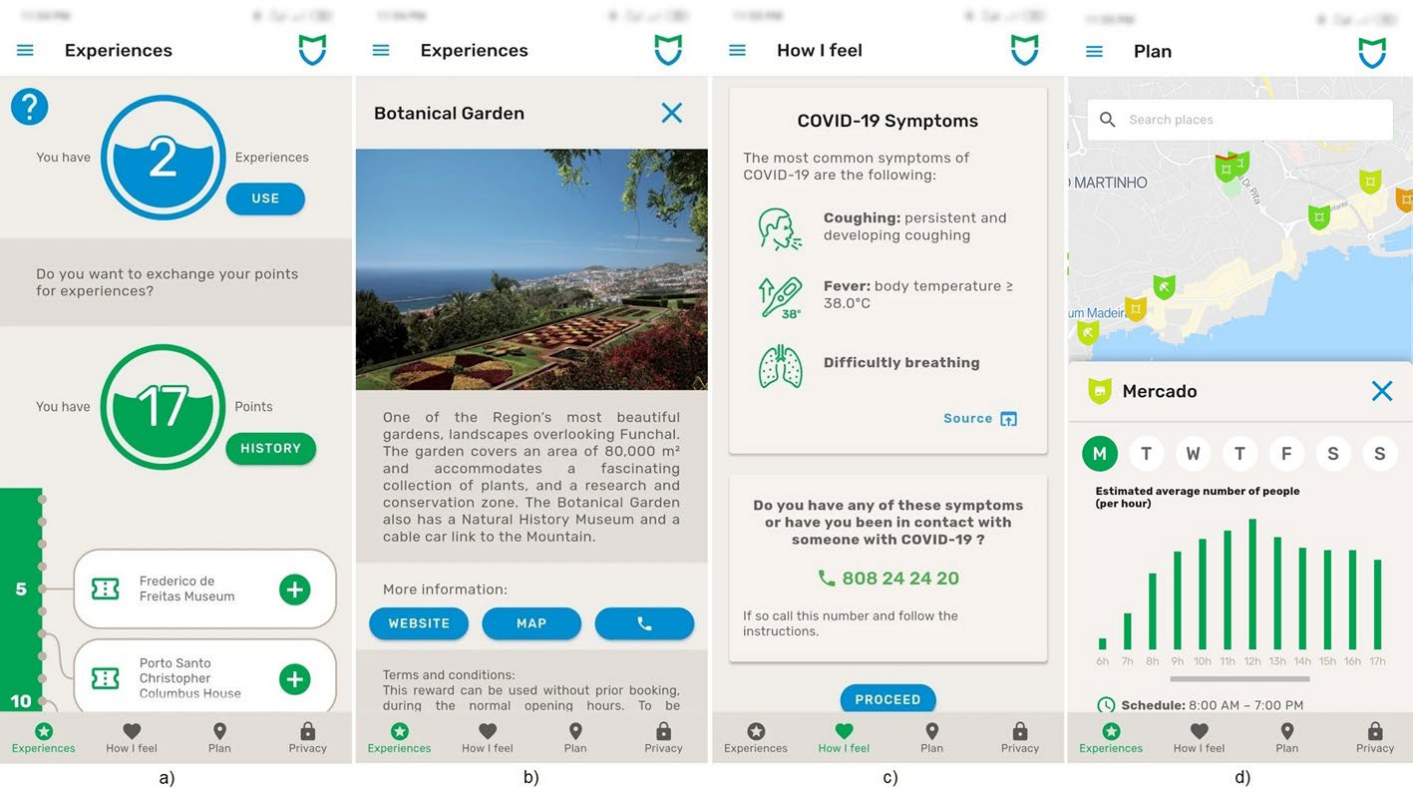
Mobile application screens [8] MadeiraSafe Mobile application screens

With many COVID-19 applications focused on digital contact tracing and Portugal already having a governmentally approved application, on our regional level, we decided to focus on the information and prevention aspect and developed a mobile application [8] that used the data gathered from the passive Wi-Fi data in POIs. The territory categorizations presented previously inform the locals and visitors about the historical and real-time occupation of the popular destinations of the island. The application was developed in partnership with the Health and Tourism authorities executing the Islands’ secure tourism plan. This plan depended on the air terminal (and harbour) PCR COVID-19 tests, and the tourists might either transfer the result of a COVID-19 PCR test to the app or pass the health check at the airports’ arrivals after that. They could also perform a free PCR test on entry and get the test result through e-mail. During the 14-day monitoring period, the tourists were inquired to fill out a voluntary epidemiological study. The application then provides the information categorized into four tabs, focusing on *Experiences*, *How I Feel*, *Plan* and *Privacy*. The focus is on the prevention and visits planning aspects. The user can collect points when visiting locations deemed safe according to the health authorities and the real-time Wi-Fi occupation detection. The information is made available to the users as depicted in Fig. 16.

a & b) Experiences—This section shows the user an overview of the current points collected and the experiences that can be exchanged for points, as well as a vertical chronological view of the visit history, besides, when clicking on a past experience the user can see its details and current occupation;

c) How I Feel—This section displays common recommendations from the health authorities with the ability to can call emergency numbers;

d) Plan—In this section, the user can scout the island in a map view and see the real-time occupancy of the POIs denoted by a shield that changes colour (green to red scale). Upon touching a POI, the application presents to the user the historical occupancy of the diverse weekdays’ and hours and presents information about the POI, such as website, address, contacts, and schedule;

Privacy (not depicted)—This section allows the user to view in a transparent manner how the information that is being presented is collected, and the ability to set notification permissions and geolocation (used to locate the user in the map.

The real-time map data and the historical occupation charts are powered by the passive Wi-Fi infrastructure that collects data anonymously in these locations. Using the territory categorization and passive Wi-Fi datasets presented in this article, the application was part of a more broad safety framework, which included testing all tourists, inquiring them to fill out an epidemiological questionnaire and status check every day for COVID-19 symptoms. The gamification component of the application that awarded users with points every time they visited a COVID-19 safe POI also motivated the tourists to perform the PCR test before their arrival via the attribution of points for that action.

Even though the application’s gamification component is outside this article’s scope, it incentivized users to focus on completing assignments and the challenges of visiting secure areas and POIs. The points earned could then be traded at no cost for experiences proposed by the regional tourism office. Also, the first time use, the application interface begins by displaying the user with opening information, which introduces the user through the four fundamental screens of the application and allows to understand the points system, the experiences, challenges and mechanics.

## Discussion and conclusion

This article shows that it is possible to use passive Wi-Fi data to categorise different locations according to their use. The use of passive Wi-Fi data has the advantage of being data-sparse and preserving privacy. In addition, we show and infer differences between 2019 and 2020 for the Wi-Fi counts’ pre and during the COVID-19 pandemic (RQ1). It is noticeable a decrease in Wi-Fi activity in the territory hexagons relative to transportation, commerce, and education for 2020. This is the direct result of the confinements and work/study from home recommendations. The territory categorisation serves as meta-data and enables a better understanding of other spacial datasets, by providing some data explicability of peaks or trends in the data.

We have shown a strong correlation between passive Wi-Fi and telecom data (RQ2), and there is the possibility of using passive Wi-Fi sensing data for crowdsensing. With the telecom data being provided on a parish level, the results show varying levels of correlations between the telecom data used as ground truth and the Wi-Fi data. Since both datasets are indicators of activity at a parish level, depending on the locations of the Wi-Fi routers. Some parishes activities are represented better than others, as the fewer the routers, the more the generalization that is being made to infer the activity of the whole parish. Despite that, the results show correlations higher than 0.7 for five of the nine parishes, going as high as 0.9. This shows that the Wi-Fi data can be used as a proxy to infer the activity in the parish that is represented in this case via the telecom cellular association changes.

In addition, we show that passive Wi-Fi data can also be used to forecast activity data (RQ3). Wherein this case, we used a subset of pre-pandemic 2019 data. Otherwise, the historical data could not predict the decrease in activity caused by COVID-19 in 2020. The data proved to be periodic, and thus the results show that the POIs can be clustered into six groups. For each group, we can successfully use methods such as VARX to forecast the trends, with train/test ratios of 60/40%, obtaining a 17% symmetric mean absolute percentage error. Based on the setup using Wi-Fi routers deployed on Madeira, we present an ecosystem that culminated with a mobile app to ensure that these datasets and results are made available to the citizens. Therefore we show that we can successfully use passive Wi-Fi data to inform, help, and prevent the spread of the COVID-19 disease in crowded places. Our approach is cheaper, easier to deploy and preserves privacy, unlike many other techniques using cameras [57, 58], cellular data [59] and mobile app tracking [60] which are typically hosted by major companies and not owned and managed by local communities.

The results that emerge from this work can be useful to decision makers to benefit, not only from updated territory data in comparison to outdated data from census, but also, the daily updates from the telecom data enable the creation of dashboards with information regarding site loads, the display of historical data, and provide data modeling more accurately to predict future situations. With more data sources to complement the decision making process, the making of policies can be more adequate to the territory in question by taking into account how the citizens move and also leveraging the mobile application to provide relevant information or non-intrusive notifications to inform the population about important aspects of the situation in question.

This analysis is not without its limitations, the data itself is limited to the dataset providers, such as the telecom data was only provided for a specific period of time, and for research purposes. To keep the data anonymous for small locations, the minimum territory division provided was the municipality, where in some larger ones, the parish granularity would have been preferred. The data sourced from OpenStreetMaps, has the limitations of the API, and how much viable the information is about the services available, which in some locations is not updated regularly, or not precise enough in comparison to other main locations, thus influencing the data analysis or results. Furthermore, the analysis presented here is limited to an island context.

Finally, future work includes fine-tuning the occupational estimation models for particular locations that do not fit the generalized modelling, validated with more inputs of ground truth. We prepare questionnaires and usage data collection on the mobile application to get the users’ feedback and act as a crowdsourcing platform to collect the users’ perception of occupation and safety in specific locations in a non-intrusive manner.

## Data Availability

The datasets analysed during the current study are available from the corresponding author on reasonable request. Nonetheless, restrictions apply to the availability of cellular telecom data which was provided under license for analysis only, and so are not publicly available.

## Footnotes

1 https://covid19.apple.com/contacttracing.

2 https://blog.google/documents/55/Android_Contact_Tracing_API.pdf/.
